# Quality by Design for the Nanoformulation of Cosmeceuticals

**DOI:** 10.3390/pharmaceutics18010062

**Published:** 2026-01-01

**Authors:** Gerardo Leyva-Gómez, Elizabeth Piñón-Segundo, Zaida Urban-Morlan, Nancy E. Magaña-Vergara, David Quintanar-Guerrero, Betzabeth Jaime-Escalante, Néstor Mendoza-Muñoz

**Affiliations:** 1 Departamento de Farmacia, Facultad de Química, Universidad Nacional Autónoma de México, Ciudad de México 04510, Mexico; leyva@quimica.unam.mx; 2 Laboratorio de Sistemas Farmacéuticos de Liberación Modificada, Facultad de Estudios Superiores Cuautitlán, Universidad Nacional Autónoma de México, Cuautitlán Izcalli 54714, Mexico; pinonsegundoelizabeth@cuautitlan.unam.mx; 3 Centro de Información de Medicamentos y Farmacia Clínica, Facultad de Química, Universidad Autónoma de Yucatán, Mérida 97069, Mexico; zaida.urban@correo.uady.mx; 4 SECIHTI Investigadores e Investigadoras por México, Facultad de Ciencias Químicas, Universidad de Colima, Colima 28400, Mexico; nancymv@ucol.mx; 5 Laboratorio de Investigación y Posgrado en Tecnología Farmacéutica, Facultad de Estudios Superiores Cuautitlán, Universidad Nacional Autónoma de México, Cuautitlán Izcalli 54740, Mexico; quintana@unam.mx; 6 Departamento de Sistemas Biológicos, Universidad Autónoma Metropolitana, Ciudad de México 04960, Mexico; 2203800268@alumnos.xoc.uam.mx; 7 Laboratorio de Farmacia, Facultad de Ciencias Químicas, Universidad de Colima, Colima 28400, Mexico

**Keywords:** cosmeceuticals, nanotechnology, Quality by Design, nanocarriers, bioactives

## Abstract

Cosmeceuticals are cosmetic formulations that are intended to alleviate skin conditions that affect its appearance and functionality. They are not considered medications but contain molecules that exert biological action on the skin beyond traditional cosmetic actions. Sometimes, the bioactives used have limitations for transdermal passage, and it has been suggested that the use of nanocarriers can increase the effectiveness of cosmeceutical products. The degree of sophistication of nanocosmeceuticals requires that safety and efficacy aspects be verified before going on the market. In this regard, the application of the Quality by Design (QbD) approach during product development ensures that products meet the consumer needs in full. This review analyzes the implementation of QbD in the development of nanocosmeceuticals, considering the main characteristics of the most used bioactive groups and nanocarriers that have proven to be ideal vehicles for topical and transdermal applications.

## 1. Introduction

Cosmeceuticals are cosmetic products that contain ingredients that are proposed to improve the health and appearance of skin, and recent approaches have focused on the design of novel delivery systems. In this way, nanotechnology has played a crucial role in cosmeceutical development; nanoformulations have revolutionized the cosmetics industry by enhancing product efficacy and addressing various skin concerns. Nanosystems, such as liposomes, polymeric nanoparticles, nanoemulsions, and solid lipid nanoparticles, are utilized in cosmetics for improved UV protection, deeper skin penetration, and extended stability of cosmetic active ingredients. These nanocosmetics offer solutions for chronological aging and photoaging, hyperpigmentation, acne, hair fall, dandruff, etc., receiving the name nanocosmeceuticals [1,2].

The global nanocosmeceutical market is expanding rapidly, with significant investments by cosmetic manufacturers. However, safety concerns have been raised regarding the potential risks posed by nanoparticles to human health and the environment. Regulatory bodies around the world are working to address these issues and establish guidelines for nanocosmeceutical manufacturing [1]. Despite the challenges, nanocosmeceuticals must be designed from the outset as products with proven safety and efficacy. The design stage must consider the complexity of nanoformulations, which are subject to inherent variations in starting materials and manufacturing methods. In this sense, the Quality by Design (QbD) approach presents itself as a serious alternative for obtaining nanocosmeceuticals with proven efficacy and low risk to consumers [3].

QbD is a systematic approach to develop and produce goods based on knowledge and risk management. This approach is widely used in pharmaceutical development to enhance product and process performance, addresses manufacturing challenges, and ensure regulatory compliance. The QbD principles include identifying Critical Process Parameters (CPPs) and Critical Material Attributes (CMAs), implementing risk management strategies, and utilizing Design of Experiments (DoE) as well as Process Analytical Technology (PAT); it is a powerful tool successfully applied to various nanocarriers, including liposomes, polymeric nanoparticles, and solid lipid nanoparticles [3].

These principles are especially valuable in the context of nanotechnology-based cosmeceuticals, where formulation complexity can lead to batch-to-batch variability, scale-up issues, and potential recalls. It outlines the desired product characteristics, including physical and physicochemical characteristics of nanocarriers (size, surface charge, encapsulation efficacy, etc.), cosmetic vehicle properties, depth and rate of penetration, safety and stability requirements, and consumer expectations. In this review, we explore the integration of QbD into nanotechnology-based cosmeceutical development. We highlight how QbD principles can enhance product design and optimize manufacturing processes, ultimately ensuring the delivery of safe, effective, and high-quality products to consumers.

This review explores how QbD principles can be strategically integrated into the development of nanocosmeceutical products to ensure their safety, efficacy, and quality by addressing the inherent complexities of nanoformulations—such as variability in materials, manufacturing challenges, and regulatory and consumer demands—and the specific biological effects that cosmeceuticals are expected to produce in the skin, beyond their aesthetic benefits. Despite the publication of several research and review articles applying QbD principles for the design of nanocosmetics, this article aims to demonstrate how QbD offers a systematic, science-driven framework for optimizing formulation design, enhancing process control, and minimizing risks in a specific category of nanocosmetics, the nanocosmeceuticals. Finally, this review advocates for QbD as a transformative approach that supports innovation while safeguarding consumer health and meeting industry standards in the rapidly evolving nanocosmeceuticals market.

## 2. Cosmeceuticals

### 2.1. Definitions

The U.S. Food and Drug Administration (USFDA) has defined cosmetics as “substances intended for application to the human body aimed at cleansing, beautifying, promoting attractiveness or altering the appearance without affecting the body physiology or functions.” Cosmetics that contain bioactive agents are known as cosmeceuticals but are not regulated by the USFDA [4].

The term cosmeceutical is a fusion of the words cosmetic and pharmaceutical, used to describe cosmetic products with defined beneficial biological activity in the skin and appendages. It was originally coined by Raymond Reed, founder of the U.S. Society of Cosmetic Chemist, in 1961. The concept was further investigated and popularized by Albert Kligman in the late 1970s. A cosmeceutical is mostly considered a skincare product because of its biological action on the skin like a drug but is regarded as cosmetic since it affects the appearance. Cosmeceuticals can restore, heal, or improve the physiological functions of skin [4,5,6,7].

Cosmeceuticals are a fast-growing segment of the personal care industry, including skin care, hair care, oral care, nail care, and lip care. Several topical cosmeceutical treatments are frequently used to improve tone, texture, and radiance of the skin and to treat or avoid wrinkles, black spots, dry skin, uneven skin tone, photo aging, hair damage, and hyperpigmentation [5,6,8]. There are other terms that are frequently used to refer to cosmeceuticals, such as bioactive cosmetics, nutricosmetics, performance cosmetics, functional cosmetics, skinceuticals, dermocosmetics, and dermaceuticals [5].

The ability of cosmeceuticals to enhance skin functioning does not only depend on its mechanism of action and concentration but also depends on the incorporation of ingredients into a topical vehicle that maintains the integrity of the active, delivers the active in a biologically appropriate form, reaches the target site in sufficient quantity to exert an effect, and properly releases the ingredient from the carrier vehicle [5,9].

Nanotechnology has enabled the development of improved cosmeceutical products with enhanced properties, like better skin penetration, targeted delivery, controlled release, increased stability, and well-enhanced occlusion effect. Nanocosmeceuticals are a class of cosmeceuticals where actives are formulated with nanotechnology, i.e., nanovehicles or nanocarriers [4,6,10].

Since the first nanocapsule-based cosmetic product composed of vitamin E was launched by the company L’Oreal in 1995 to combat skin aging, many novel and submicron-sized nanostructured materials have been used in the delivery of cosmeceutical formulations, comprising nanocapsules, nanospheres, dendrimers, nanoemulsions, liposomes, solid lipid nanoparticles, nanostructured lipid carriers, nanocrystals, niosomes, ethosomes, cubosomes, ultrasomes, photosomes, transfersomes, silver or gold nanoparticles, and fullerenes [4,8,11,12,13,14].

Nanocosmeceuticals are widely employed in sunscreens, moisturizers, hair care preparations, antimicrobials, antiaging and antiwrinkle creams, skin cleansers, as well as lip, nail, and oral care products [8,12,13]. Some comprehensive reviews covering nanocosmeceutical products that are available on the market are worth reading [11,13,14,15,16,17].

### 2.2. Bioactives

Cosmeceuticals are cosmetic products with added active ingredients that offer biological activities, going beyond simple cosmetic enhancement. These ingredients can have pharmaceutical-like effects on the skin. The biological activities considered in cosmeceuticals are antioxidant, anti-inflammatory, antipigmentary, antiacne, antiwrinkle, promotion of cell regeneration, moisturization, microbiome restoration, and hair growth, as the most relevant.

Actives used in cosmeceuticals can be obtained either from a natural source (bioactives) or by chemical synthesis. Consumers consider nature-based products safer and less toxic than those obtained by chemical synthesis [18]. Some plant-based bioactive compounds have therapeutic properties, including moisturizing, reviving, antiaging, UV protection, and the prevention of skin-related disorders [8]. Plant-based bioactive compounds are highly desirable active ingredients in the cosmeceuticals business [19]. However, not only has the obtaining of bioactives from plants been explored, but a search for cosmeceutical bioactives in marine sources, such as algae, microalgae, and other marine organisms, has been carried out; these sources are rich in bioactive compounds, including polysaccharides, peptides, fatty acids, antioxidants, and minerals, offering various benefits for skin health, such as antiaging, anti-inflammatory, and hydrating properties [20]. Another interesting group of bioactives are biomimetic peptides. Biomimetic peptides have a wide range of applications, including botox-like effects, cell proliferation activity, protein synthesis promotion, antipigmentation, anti-inflammation, and hair growth, among others. Biomimetic peptides can be sourced from natural or synthetic origins. Plant-based sources include soy, oats, and rice, while animal sources include venoms, membrane proteins, etc. [21,22].

## 3. Nanocarriers for Skin Delivery

The topical administration for many active molecules in cosmeceutical products is limited due to the skin’s Stratum Corneum (SC) acting as a barrier, limiting most molecule penetration [23,24].

**Figure 1:**
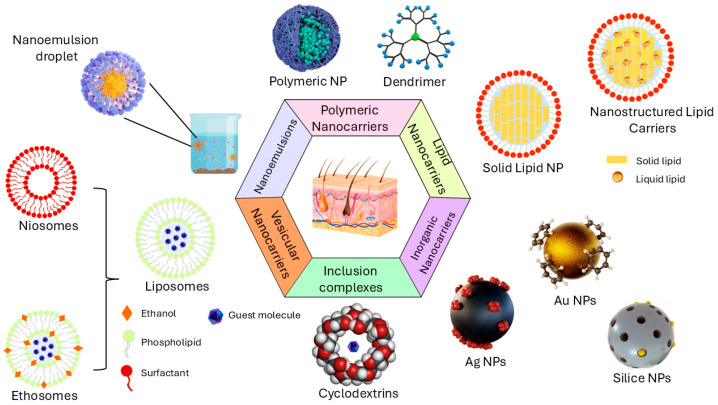
Schematic nanocarriers for skin delivery.

Nowadays, novel nanocosmetic formulations have been designed to optimize the delivery of active ingredients through the skin using nanocarriers. These Nanocarriers (NC) are generally classified as colloidal structures with a mean diameter of fewer than 10–100 nm but, in some cases, from 100 to 500 nm [25]. NC can be obtained from several materials, such as lipid, polymeric, inorganic, and surfactant-based (Figure 1), forming different structures such as liposomes, micelles, nanotubes, inorganic nanoparticles, dendrimers, and nanoemulsions [16,26,27].

The use of nanocarriers helps to improve the stability of biomolecules in cosmeceutical formulations, promotes or retards the skin permeation of molecules, provides the most controlled release of actives, improves hydration through film formation on the skin, and shows less side effects than their conventional administration. Nanotechnology-based formulations have been used in different beauty and skincare products, such as sunscreens, deodorants, perfumes, hair care, and dental products.

In the conventional pathways of molecular penetration across the SC (intercellular, transcellular, appendageal), some mechanisms enhance penetration of nanocarriers across the SC [28,29], such as skin hydration, system deformability, disruption of the SC, surface charge, and particle size [24,29].

Nanocarriers show high adhesiveness to superficial SC, forming an occlusion layer on the skin surface, increasing the skin’s hydration, and facilitating the delivery of active molecules. Also, nanocarriers can change shape or structure in external conditions, such as pH and temperature, and this deformability increases skin penetration [30,31].

The presence of nanocarriers can disturb the structure of the SC [29,31,32]. In this context, ethanol in some NCs improved skin penetration and bioavailability, modifying the structure of the lipophilic or keratinized domains in the SC [26].

The surface charge and the particle size of nanocarriers can influence skin penetration, adherence, and degradation [33]. The cationic nanocarriers interact with the negative charge of the skin surface via electrostatic interactions and promote transdermal permeation but exhibit higher cytotoxicity. On the other hand, the adsorption of negatively charged nanocarriers occurs via endocytosis [31,32]. Smaller molecules (<500 Da) diffuse through the skin faster than larger molecules [34].

### 3.1. Polymeric-Based Nanoparticles

Polymer Nanoparticles (PNP) can be prepared by biocompatible polymers obtained from natural or synthetic sources [35,36,37,38,39]. The principal kinds of PNP include nanocapsules, nanospheres, polymeric micelles, and nanohydrogels. The preparation methods consist of the polymerization of monomers and the dispersion of preformed polymers [40,41].

Various groups reported the potentiality of chitosan nanoparticles to encapsulate active molecules [42], such as α- and β-arbutin in melasma treatment [43,44], α-tocopherol (vitamin E) with antioxidant activity [45], functional oils [46], and nicotinamide or clindamycin in acne vulgaris treatment [47,48], which showed better therapeutic efficacy, stability, and controlled release properties compared to the free drug.

### 3.2. Lipid-Based Nanoparticles

Lipid Nanoparticles (LNPs) are colloidal systems composed of a lipophilic core stabilized in a water phase using a monolayer of emulsifiers [49,50]. These carriers are fabricated using biodegradable and biocompatible lipids (triglycerides, glycerides, fatty acids, and waxes), emulsifiers (phospholipids, poly(ethylene glycol)-based (PEGylated) surfactants), and water. Due to their structural characteristics, they can encapsulate active ingredients that are poorly soluble in water [51].

**Figure 2:**
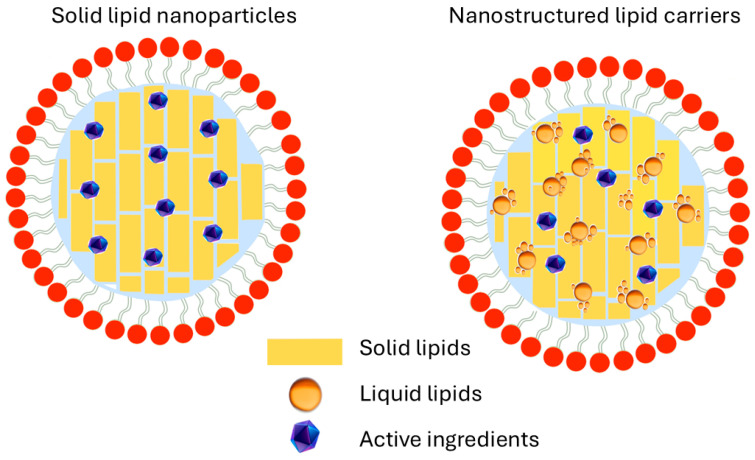
Schematic illustration of the structures of SLNs and NLCs.

Several types of LNPs are currently used in cosmetic technology; these include Solid Lipid Nanoparticles (SLNs), Lipid Nanoemulsions (LNEs), and Nanostructured Lipid Carriers (NLCs) [52]. The main difference between them lies in their core composition (Figure 2). Many methods to produce LNPs have been developed, including high-pressure homogenization [53], the microemulsion technique, and emulsification-solvent evaporation. Recently, SLNs and NLCs have been prepared via green procedures such as ultrasonication [54], ultrafiltration [55], flocculation with surfactants [56], and the supercritical fluid method [54,57,58,59].

SLNs are produced by replacing the liquid lipid (oil) of an o/w emulsion with a solid lipid or a blend of solid lipids. The lipid particle is solid at room and body temperature and stabilized by adding surfactants [60,61].

Nanostructured Lipid Carriers (NLCs) are the most recent generation of lipid nanoparticles, whose composition is derived from a combination of solid and liquid lipids. The nanoparticles are stabilized in an aqueous medium using at least one surfactant in a concentration ranging from 0.5 to 5% *w*/*w* [51]. Nanoemulsions are formed by the addition of two immiscible phases (oil and water) through the addition of a suitable emulsifier. The droplet size of nanoemulsions is typically between 20 and 300 nm, with a narrow particle size distribution.

Studies have shown the ability of SLNs and NLCs to incorporate lipophilic or poor water-soluble molecules, such as carotenoids [62], polyphenols (sesamol, resveratrol) [63], essential oils [64,65], and other active molecules (tocopherol, retinoids, and coenzyme Q10) [66,67,68,69], with potent antioxidant activity, depigmenting and photoprotective properties, and antimicrobial activity. These reports demonstrate that using SLNs and NLCs as encapsulants increases the stability of molecules sensitive to oxidation or hydrolysis, reducing degradation compared to aqueous dispersion.

Samprasit et al. [70] describe the comparative study of resveratrol loaded in Nanostructured Lipid Carriers (NLCs) and Nanoemulsion Gels (NEGs), exhibiting different physical properties and antioxidant activity depending on the composition. The NEGs had smaller particle sizes, higher resveratrol content, faster release, and greater stability than the NLCs. However, the NLCs demonstrated more antioxidant activity and less cytotoxicity than the NEGs.

### 3.3. Miscellaneous Nanosystems

**Figure 3:**
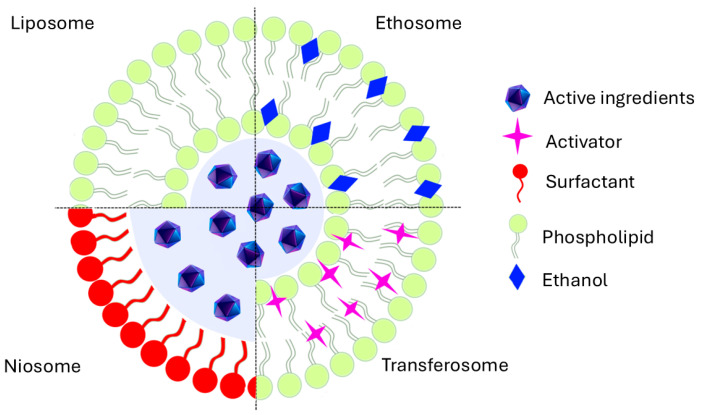
Structural features of vesicular nanocarrier systems.

Liposomes are sphere-vesicular carriers composed of one or more phospholipid bilayers where a lipidic membrane surrounds an aqueous volume. To improve the bilayer features of the liposome, cholesterol can also be used. Other types of vesicular systems that share structural similarity with liposomes but differ in composition include niosomes (non-ionic surfactant-based vesicles), ethosomes (ethanol-core vesicles), and transfersomes (transdermal optimized ultra-deformable vesicles) (Figure 3). Ethosomes are flexible multilayer vesicles composed of phospholipids, water, and a high ethanol concentration (20–40%) [71]. Compared with liposomes, ethosomes offer better features for efficient delivery of cosmetics to the skin. Niosomes are small vesicles composed of non-ionic surfactants. In cosmetics and skincare, they can improve the bioavailability of poorly absorbed ingredients, effectiveness, penetration, and stability.

Vesicular nanocarrier systems have been widely reported in cosmetics and skincare products. Moreover, they have been applied in a variety of skin pathologies, such as acne vulgaris, psoriasis, atopic dermatitis, skin cancer and skin infections, melasma, hyperpigmentation [72,73,74], and in some skin care products for antiaging and UV protection [75,76,77].

In order to improve their stability and skin penetration, vitamins have been encapsulated into liposomes [78,79,80], demonstrating that the composition of the liposome improves the interaction with keratinocytes and fibroblast membranes, increasing the amount of vitamin C crossing the skin [78].

Inorganic nanoparticles are suitable additives for improving a cosmetic formulation’s rheology and optical properties. Some nanoparticles, such as titanium dioxide and zinc oxide, silver and gold nanoparticles, and silica nanoparticles, are used in cosmeceuticals [81].

Titanium dioxide (TiO_2_) and zinc oxide (ZnO) nanoparticles are employed as UV filters in sunscreens because of their ability to block ultraviolet radiation from sunlight: UVA (320–400 nm) and UVB (290–320 nm) [82,83,84]. Silver (AgNP) and gold (AuNP) nanoparticles have antibacterial and antiseptic properties; therefore, they are used as active ingredients in preparing skincare products, toothpaste, and deodorants. Also, their antifungal activity is used in nail polish to treat fungal toenail infections [85,86].

Mesoporous silica is a porous form of silica consisting of a hexagonal array of nano-sized pores. These empty pores can incorporate active molecules and release them in controlled ways [87]. In a recent study, mesoporous silica was used to encapsulate octyl methoxycinnamate (OMC), the organic sunscreen, and the resulting formulation showed nanoparticles can delay OMC release and provide 57% better UV protection than the free OMC [88,89].

In Table 1, we present some characteristics of each category of nanosystems and considerations for QbD implementation.

## 4. Considerations for Implementation of QbD in the Nanoformulation of Cosmeceuticals

### 4.1. Quality by Design Framework

One of the main goals of the cosmetic industry is to consistently produce formulations that ensure quality, safety, and efficacy to meet the changing requirements of the consumer. The cosmetic market is characterized by dynamism and short product development periods, with a wide variety of suppliers or producers. Under this scenario, cosmetic enterprises should work efficiently if they want to have presence in the market and be competitive. This means that, during every step of the manufacturing process, quality must be maintained. Quality has stood as the pillar to fulfill consumer satisfaction through a product, service, or process. In this regard, quality activities must be oriented to detect quality problems in the early stages of production and not just detect failures in the final product. The golden quality statement promotes precaution rather than correction of problems, and to achieve this goal, quality must be built and maintained not only in the product but also in all the manufacturing steps through proper planning. This idea was first introduced by Joseph Moses Juran, and now, it is stated that QbD refers to the fact that quality should be designed into a product, and most quality issues are related to the way a product was designed in the first place [90,91]. In fact, QbD was first encouraged by the FDA among pharmaceutical producers to adopt this quality approach in drug product development, manufacturing, and regulation [91]; also, the USFDA emphasized that increment of final testing does not necessarily improve product quality. The importance of QbD in industry lies in that it is a structured methodology for product development, offering a quality system framework that harmonizes lifecycle and regulatory obligations, enhances process knowledge, and prioritizes consumer safety [92].

Then, in 2005, QbD consolidated with the publication of ICH Q8 (Pharmaceutical Development), ICH Q9 (Quality Risk Management), and ICH Q10 (Pharmaceutical Quality System); all these documents provide important directions for the rigorous pharmaceutical industry [91,92]. Nevertheless, the principles of QbD can be applied to cosmetic development due to the necessity of producing high quality products that meet regulatory requirements and user expectations. Implementation of QbD elements in a cosmetic enterprise can exert remarkable advantages because, nowadays, it is focused on sustainable processes to reduce waste, energy, and material usage in the production of cosmetic products [93].

### 4.2. Elements for QbD Implementation

ICH Q8 contains the definition of QbD as “a systematic approach to development that begins with predefined objectives and emphasizes product and process understanding and process control, based on sound science and quality risk management” [94]. This definition can be adapted to the cosmetic industry under the premise that quality cannot be assessed into products, but rather, it should be built in them from the design step [95].

It is widely accepted that the elements of QbD are [90,91,92,93,96] as follows:

1. Determination of Quality Target Product Profile (QTPP);
2. Critical Quality Attributes (CQAs) assessing;
3. Risk assessment and determination of Critical Material Attributes (CMAs) and Critical Parameter Process (CPPs);
4. Design space by Desing of Experiments (DoE);
5. Control strategies;
6. Continuous improvement and Lifecycle management.

### 4.3. Determination of QTPP

This is the foundational step in QbD, which involves a prospective summary of predefined quality characteristics of the final product. In general, some aspects to consider include the following:

- Cosmetic form, anatomical place of application, intended use;
- Container closure system and quantity of product per application;
- Aspects affecting permeation/retention in skin layers: dissolution, solubility, pKa, log P, molecular weight;
- Criteria of cosmetic product quality: solubility, stability, safety, active molecule release.

As was mentioned above, the definition of the QTPP allows the establishment of the predefined characteristics of the final product. Talking about nanoformulations focused as a cosmeceutical, the QTPP should be built as a prospective outline that includes characteristics centered on quality per se, safety, and efficacy. The QTPP is usually presented as a list or table of Quality Attributes (QAs), followed by the target values of each attribute (numerical or categorical) and its justification in case of being considered CQAs.

In cosmeceutical nanoformulations, the QTPP could be defined in the same way as nanoformulations for dermal drug delivery [97]. In Table 2, we address the elements of the QTPP that correspond to the cosmetic vehicle; this means the formulation in which the nanoparticles (with the cosmeceutical inside) are incorporated, rarely dosed just as are obtained from the different preparation methods because they do not comply with the appropriated characteristics of a cosmetic product.

In Table 3, we present the elements of QTPP that are related to the nanoparticles in which cosmeceuticals are included. The objectives of nanoencapsulation are protection from harsh environments, skin irritation reduction, stabilization enhancement, promotion or retarding of bioactive penetration, and controlled release, among others.

### 4.4. Considerations for Assessing Critical Quality Attributes in Nanocosmeceuticals

Defined by ICH Q8 R2, a CQA is “a physical, chemical, biological, or microbiological property or characteristic that should be within an appropriate limit, range, or distribution to ensure the desired product quality”. The importance of defining the CQAs is based on the necessity of knowing the impact of quality features on the efficacy, safety, and quality of the product [91]. CQAs are derived from the QTPP, which outlines the desired product performance characteristics; in nanocosmeceuticals, the objective of the identification of CQAs of a product is to ensure the product’s quality, safety, and efficacy. One of the elements that must be considered a CQA is particle size, primarily because defining a product as a nanocosmeceutical requires specific size characteristics. The UE defines “nanomaterial” in cosmetic products as an intentionally manufactured, insoluble or biopersistent material that has one or more external dimensions, or an internal structure, on the scale of 1 to 100 nm [105]. However, sometimes in the cosmetic and pharmaceutical fields, the size of some nanosystems can be considered within a range between 1 and 1000 nm by “nano” prefix definition [106,107,108], but the preferred size is between 400 and 700 nm to have good penetration capability [109]. Determining a target size as a CQA has implications for both efficacy and safety. To date, there is no consensus or sufficient evidence to generalize how the particle size of a nanosystem impacts the release, absorption, or retention of a substance through the skin [102,110,111]. Relevant studies must be conducted for each case, and based on the evidence, the optimal particle size and distribution must be determined for each application. In some cases, a smaller particle size could ensure good penetration, but in others, it could increase adverse effects. In any case, a narrow distribution will always be desirable to reduce variability in the permeation of the active ingredient or its interaction with the skin. Furthermore, the speed and quantity at which the bioactive permeates through the skin result in potential CQAs because these are attributes directly related to the product’s efficacy, safety, and performance. While quantity and penetration capacity may be properties associated with particle size [110], the rate at which the active ingredient is released is a more complex process that requires an analysis based on the specific needs of the desired biological action and, in the case of participatory release, the physicochemical properties of the matrix and its interaction with the skin.

### 4.5. Application of Risk Analysis and Evaluation Tools

Through risk assessment, it is possible to identify the CMAs and CPPs that have significant impact on product CQAs. This assessment is performed based on a scientific foundation as well as efficient benefit to the cosmetic consumer, which is commonly executed in the initial stages of product development. Risk assessment is a process to organize information that supports decision-making within a risk management framework. This process includes identifying hazards and conducting an analysis and evaluation of risk associated with exposure to those hazards [96]. Risk assessment can be divided into three phases: risk identification, risk analysis, and risk evaluation. Table 4 shows the objective of each phase and tools for its implementation; a comprehensive discussion of these tools during the design of nanocosmeceuticals is presented below.

CMAs are the properties of an input material and can be physical, chemical, biological, or microbiological, which should be within an appropriate limit or range to ensure the desired quality. CPPs consist of a group of parameters checked before or in a process that significantly impact purity, appearance, and yield of final product.

According to QbD, CMAs and CPPs can slightly vary within some values or limits corresponding to the design space; without a significant change to a CQA, then the quality of the final product will meet the QTPP. CQAs are determined by CPPs and are identified using risk assessment from a list of potential parameters.

As previously mentioned, the regulations governing cosmeceutical nanoformulations align with those applicable to nanocosmetics. The risk analysis of these formulations considers both their nanometric properties and their systemic activity. The most common risk analysis is the Ishikawa diagram and the Failure Mode and Effects Analysis (FMEA).

The Ishikawa diagram, also known as a cause-and-effect or fishbone diagram, is a visual tool used to identify the root causes of a problem. It organizes potential causes into categories, such as people, methods, materials, equipment, and environment [112]. On the other hand, Failure Mode and Effects Analysis (FMEA) is a structured methodology used to identify products, processes, or system failures and analyze their causes and consequences.

FMEA aims to proactively mitigate risks by preventing failures or reducing their impact before they occur [112]. FMEA teams examine how a product, process, or system could fail to meet its intended function. The analysis involves assessing the effects of these failures, which may include risks, harm, defects, or waste. Additionally, the process of risk mitigation helps identify potential failures early, allowing organizations to take preventive measures to eliminate or minimize risks [112]. To assess risk, FMEA often employs a Risk Priority Number (RPN), which is calculated by multiplying three factors on a scale of one to ten: severity (S), occurrence (O), and detection (D) [113]. By considering these factors, organizations can prioritize which risks need to be addressed and improve their reliability and safety. An example of FMEA for a nanocosmeceutical product is shown in Table 5.

Related to cosmeceutical nanoformulations, the primary risk is their small size, which enhances their mobility and ability to cross biological membranes. This allows them to access cells and tissues that larger particles cannot reach [114]. Additionally, toxicity and chemical reactivity at the nanoscale tend to increase due to changes in the fundamental properties of the substances involved [17]. These nanoscale particles can enhance transport, absorption, bioavailability, and the prolonged effects of products, potentially triggering a more significant biological response [114]. The increased chemical reactivity of these particles, stemming from alterations in their fundamental properties, can also make them unstable during storage, leading to changes in the characteristics for which they were originally designed [17].

Another significant risk is occupational exposure to nanoparticles, which can occur through three main routes: inhalation, ingestion, and topical application. Inhalation is the most common route, followed by ingestion and topical application [17]. Continuous exposure to these products may accumulate in tissues and organs, potentially resulting in severe damage. Manufacturers are responsible for ensuring that product labels accurately reflect all contents and alert consumers to potential hazards. This practice is crucial for providing consumers with the correct information [115].

Nanoformulations can also reach the environment through manufacturing or after consumer use. Their persistence and interaction with the environment depend on their ability to disperse into various media such as air, water, and soil as well as their mobility and stability. This can pose serious environmental concerns.

### 4.6. Design Space by DoE

The ICH Q8 definition of design space is “the multidimensional combination and interaction of input variables (e.g., material attributes) and process parameters that have been demonstrated to provide assurance of quality” [90,93].

DoE is an organized approach to analyzing the relationship between process factors and their outputs. Factors can be changed systematically according to a pre-specified design. The ICH Q8 definition of design space is “the multidimensional combination and interaction of input variables (e.g., material attributes) and process parameters that have been demonstrated to provide assurance of quality” [90,93].

Every parameter changed within the design space is not subjected to regulatory notification; on the contrary, movements outside this space would normally be classified as deviations and cause a regulatory post-approval change. Moreover, design space is determined by implementing the design of experiments, which are systematically elected and assessed by doing changes in the input factors to screen and optimize CMAs and CPPs. In practice, graphs and plots are useful to represent the design space, such as surface-response curves or contour plots, as well as a linear combination of parameter intervals, equations, and models.

The design of experiments includes the fundamental principles of randomization, replication, blocking, orthogonality, and factorial experimentation. Randomization prevents distortion due to experimental bias, and replication reinforces this by repeating the experiment to increase precision and decrease noise. Blocking restricts known nuisance factors to improve precision. Additionally, orthogonality requires independent factor effects that result from independent variations in the factors. Finally, factorial experimentation estimates the effects induced by each factor and factor combinations through a geometric and orthogonal construction of factor variations [116].

The selection of an appropriate experimental design will depend on prior knowledge of the study universe, the study objectives, the number of factors and their interactions, and the available resources to be analyzed [117]. This will impact the statistical validity and effectiveness of each design [118]. In formulation development, DoE is categorized into screening and optimization designs.

Screening designs are useful at the beginning of the DoE to select critical input factors. They are typically linear response surface models, often including Full Factorial Designs (FFD), Fractional Factorial Designs (F-FD), and Plackett–Burman designs (PBD).

Optimization designs can be derived from screening designs and are intended to have a better understanding of the influence of factors and levels on response variables, with a greater number of experiments but with a lower number of inputs [117]. Optimization designs can be built using a simplex optimization strategy as a stepwise approach to surrounding the optimal conditions in a geometric figure representation with (k + 1) corners, where k equals the number of variables in a k-dimensional experimental domain [119]. However, most optimization designs are built with Response Surface Methodology (RSM) to determine an exact optimum. The RSM is modeled using a polynomial function that contains quadratic terms, allowing for the graphical illustration of the relationship between different experimental variables, a very convenient graphical representation for decision-making [119]. The most used optimization designs are three-level full factorial designs, Central Composite Designs (CCD), and Box–Behnken Designs (BBD). The selection of a DoE for the development of cosmeceutical nanoformulations will largely depend on our knowledge of state of the art, our understanding of the frontiers of knowledge, similar developments for other products or the scope of different technologies, and, most importantly, the time, resources, and infrastructure available. Table 6 includes a resume of DoEs and characteristics to set up during design space in nanocosmeceutical development.

### 4.7. Implementation of Control Strategies

**Figure 4:**
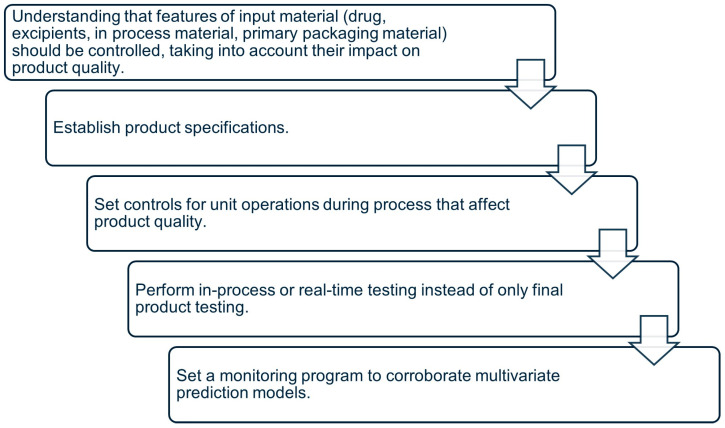
Relevant aspects to perform control strategies during product development.

Data collected from the design of experiments finishes with the control strategy [91]. According to ICH Q8 (R2), control strategy is defined as “a planned set of controls, derived from current product and process understanding, that ensures process performance and product quality” [94]. It is possible to classify three levels of control to explain and apply in the construction of the strategy. Briefly, level 1 refers to controls using automatic engineering to check and monitor CQAs in real time of the output materials. Attributes are also monitored for input materials, and parameters of the process can be adjusted to ensure that CQAs are consistent with the limits of acceptance. Level 2 is an approach that reduces the control of end-product testing and is flexible on the material attributes or process parameters within the design space. Finally, level 3 is the control commonly applied in the pharmaceutical industry, and this strategy performs extensive end-product testing with rigorous evaluation of material attributes and process parameter compliance. The implementation of a control strategy can include the aspects depicted in Figure 4.

Nanoformulation of cosmeceuticals may not yield a sufficiently robust product, even after a broad design space exploration [117]. This is due to the high reactivity of materials in nanometer size. A high surface area favors flocculation and coalescence, with the potential for phase separation through sedimentation or cremation.

CMAs and CPPs are guaranteed within certain established limits during the control strategy. The control strategy analyzes critical variables, establishes operational limits, and monitors potential risks to prevent incidents; Ref. [120] suggests including the following in a control strategy:

- Controls on material attributes (raw materials, general reagents, packaging materials, among others);
- Controls on active substances (integrity, purity, quality, and performance);
- Controls implicit in the design of the manufacturing process (unit operations, obtaining the cosmeceutical nanoformulation, sequence of purification steps, packaging);
- In-process controls (PAT monitoring).

Controls on material attributes may include descriptions of the molecular formula, weight chemical structure, purity, stability, and potential stereochemical changes and the quality and control required as well as the level of compliance allowed. The requirements and initial control over material attributes are critical conditions that enable the adequate performance of manufacturing processes or limit the achievement of formulation objectives. Particularly for the manufacture of cosmeceutical nanoformulations, changes in purity, molecular weight, or molecular presentation (acid, base, salt, or degree of hydration) will result in an increase in the average particle size, an increase in PDI, and a variation in the zeta potential beyond specifications. The possible deviations described are attributed to a change in the solubility of the raw materials [120].

Control strategies for active ingredients (such as collagen, elastin, vitamins, hyaluronic acid, and Coenzyme Q10, among others) are essential, as variations in integrity or purity can result in deviations from the product’s quality standards for functional performance. Rejection of an active ingredient due to non-compliance with quality criteria results in significant financial losses; it is typically the most expensive ingredient, along with the associated manufacturing processes.

Control strategies for the manufacturing process, as outlined in ICH M4Q [121], include a flow diagram of the manufacturing process that describes a sequential procedure narrative, along with quantities of raw materials, solvents, equipment, and operating conditions that represent a commercial batch. The flow diagram identifies critical steps and process controls.

The production of cosmeceutical nanoformulations can include critical steps such as precipitation and coacervation, which are highly influenced by speed- or frequency-dependent dispersion systems. For some nanoformulations, sequential purification steps are necessary to remove residual raw material components that do not constitute the cosmeceutical. Purification by centrifugation is a critical step that can compromise stability by increasing the average particle size, Polydispersity Index (PDI), or zeta potential. Therefore, the potential low robustness of cosmeceutical nanoformulations requires control based on monitoring particle size, population distribution, determination of zeta potential, and possibly morphology [122].

In-process controls are indispensable and critical strategies to ensure proper process performance and product quality. Traditionally, industrial manufacturing batches of cosmeceutical nanoformulations can range from one to hundreds of liters with short unit operations. PAT monitoring is intended to measure, in real time during manufacturing, critical quality attributes. Analyzing the feasibility of implementing PAT in the manufacture of nanocosmeceuticals, based on the premise that the most relevant CQAs could be particle size and encapsulation efficiency, the current analytical technologies for each are DLS and HPLC, respectively. For the former, it is necessary to transition from at-line or on-line analysis to some control techniques that may include real-time Dynamic Light Scattering with non-destructive sampling or Spatially Resolved Dynamic Light Scattering based on Fourier Domain Low-Coherence Interferometry (used in the Optical Coherence Tomography technique) for monitoring in closed packages [123] and, in addition, reflectance, emission, and transmission analysis using Fourier Transform Infrared Spectroscopy. These techniques have the advantage of being real-time without significant travel time, contamination from handling, or sample loss. They can be automated and fed with critical quality attributes to shut down the operation or alert in the event of a deviation.

The final aspect to consider is the continuous evaluation of CPPs and CMAs that should be within the lower and upper limits established to ensure reproducibility. A control strategy is always necessary to measure the impact of process parameters and attributes that affect quality; then, an assessment of reproducibility is paramount in industrial processes. To measure the reproducibility of a process, we can use the capability index (CpK), which considers upper and lower limits of a specification and the standard deviation. Indeed, the control space should be contained within the design space; the smaller the control space, the greater the robustness of the process.

Implementing control strategies focuses on identifying, preserving, and monitoring critical quality attributes to maintain process robustness within established ranges, thereby ensuring product quality [97].

### 4.8. Continupous Improvement and Lifecycle Management

The quality system described in ICH Q10 suggests continuous improvement of manufacturing processes based on scientific knowledge to promote a stronger product lifecycle. Continuous improvement is informed by product knowledge and the history of resolved process incidents. It begins in the product’s preformulation, development, and commercial life stages. Particularly in the commercial stage, adjustments to process performance, control strategies, and robustness enhancement are derived through adaptations to unit operations, equipment, facilities, and personnel training [124].

Moreover, continuous improvement of product quality throughout its lifecycle is essential, and organizations are encouraged to implement innovative tools to support this endeavor. Also monitoring process performance is necessary to confirm consistent quality results, which represents an important task of every company to obtain experience and knowledge from manufacture that can contribute to method or process development [90,91].

The lifecycle of cosmeceutical nanoformulations can also be informed by information from the manufacture of other similar products in the nanometer range, with similar compositions but different applications, as well as new production technologies and quality assurance of nanomaterials related to approval and safety regulations.

Improving the product lifecycle relies on broad, in-depth, and detailed scientific knowledge of the product itself. Therefore, knowledge management and systematization will allow for sequential analysis from different perspectives. Specialized knowledge of cosmeceutical nanoformulations will strengthen the quality system when implemented periodically in process development, technology transfer, and change management activities. Equally important is the transmission of knowledge about the design space and the product lifecycle to standardize basic understanding of the flow of communication during goal setting, particularly in leadership.

Facilitating the dissemination of knowledge across different areas and industrial plants can lead to better anticipation of deviations and, consequently, greater control over the product lifecycle and more accurate prediction of the temporal response of product quality. This is particularly true for cosmeceutical nanoformulations, which may involve manufacturing processes with low robustness or are also described as dispersed systems with a tendency toward instability when in solution.

Lifecycle management of cosmeceutical nanoformulations can be tailored to enhance functional performance while maintaining enhanced biosafety. The small size of nanomaterials and their variations can lead to different functional performances and varied biological interactions, potentially posing health risks.

**Figure 5:**
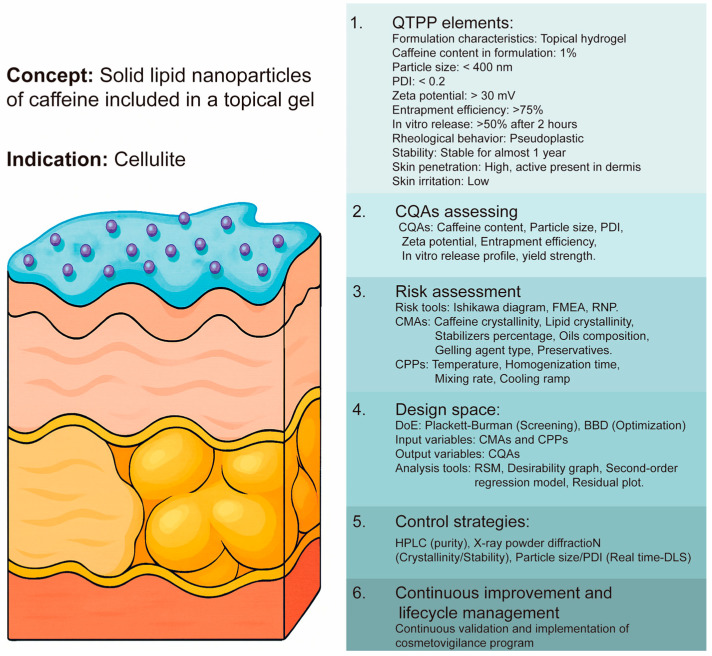
Example of QbD implementation steps and elements during the design of a nanocosmeceutical.

A robust initial control strategy for nanocosmeceuticals should tie the selected CPPs—such as particle size distribution, Polydispersity Index (PDI), and zeta potential—to real-time in-process monitoring (e.g., dynamic light scattering, FTIR spectroscopy) and well-defined finished product specifications that ensure stability and functional performance. By establishing operational limits for critical steps like precipitation, coacervation, and purification and linking them to material and active ingredient quality attributes, deviations can be detected early and mitigated. Continued lifecycle management, aligned with ICH Q10 principles, requires periodic verification of these CPPs through knowledge transfer, change management, and cross-plant communication, ensuring that process robustness and product quality are sustained and continuously improved throughout commercial manufacturing. A schematic of a complete process of implementation of QbD during nanocosmeceutical design is presented in Figure 5.

## 5. Conclusions

In the development of nanocosmeceuticals, the implementation of the QbD approach emerges as a comprehensive and forward-thinking strategy to optimize formulation, manufacturing, and quality control. The identification of critical quality attributes, critical process parameters, and critical material attributes is crucial during both stages, nanoparticle preparation and nanocosmeceutical manufacturing, to ensure product consistency, minimize batch-to-batch variability, and anticipate potential risks throughout the lifecycle. Control strategies oriented to keep physical properties under control, such as size, matrix degradation, impurities, etc., must be rigorously defined and validated to guarantee functional performance, efficacy, and consumer safety. Finally, QbD is a powerful tool that provides a scientific and regulatory framework that enhances quality in this sophisticated cosmetic delivery system.

## Figures and Tables

**Table 1:** Characteristics and considerations for QbD implementation for the different types of nanosystems.

Nanosystem	Characteristics	Considerations for QbD Implementation
Polymeric nanoparticles	Encapsulate lipophilic or poor water-soluble bioactives Generally, size > 100 nm Modulable superficial charge	High feasibility of QbD implementation; CQAs should be related to physicochemical properties and loading capability. Manufacturing process is highly controllable and control strategies viable to implement. The use of biopolymers or GRAS polymers guarantees fewer possibilities of regulatory restrictions.
Lipid-based	Encapsulate lipophilic or poor water-soluble bioactives High skin lipid compatibility Generally, size > 100 nm High occlusive effect	Very high feasibility of QbD implementation; CQAs should be related to physicochemical properties, stability, and loading capability. Manufacturing process is highly controllable and control strategies viable to implement. The use of GRAS lipids increases safety for consumers. Low possibilities of regulatory restrictions.
Vesicular nanocarriers	Encapsulate lipophilic or poor water-soluble or water-soluble bioactives High skin lipid compatibility Generally, size < 100 nm High skin penetration	Very high feasibility of QbD implementation; CQAs should be related to physicochemical properties, stability, and loading capability. Manufacturing process is highly variable but controllable and control strategies viable to implement. In the case of liposomes, it has more than 40 years on the market, with high consumer acceptability.
Inorganic nanoparticles	Used as-is or with adsorbed bioactives Generally, size < 100 nm High skin penetration	Low feasibility of QbD implementation; CQAs should be related to physicochemical properties, safety, and stability. Manufacturing process could be complicated to control; physical properties are highly dependent on synthesis route and precursors. Safety concerns related to skin bioaccumulation.

**Table 2:** Examples of QTPP elements related to cosmetic vehicles in nanocosmeceuticals.

QTPP Element	Example of Target Parameter That Could Be Used	Characteristic Impacted	Considerations
Cosmeceutical dose strength	% *w*/*w* IU	Efficacy Safety	The formulation should contain the cosmeceutical in an effective concentration based on the information of the efficacy studies.
Type of cosmetic vehicle (where the nanoformulation could be included)	Emulsion Gel Lotion Ointment Emulgel Suspension	Quality Efficacy Safety	The type and composition of cosmetic dosage form impact the sensorial profile and consumer acceptance and also influence the permeation rate of the cosmeceutical [98]. Similarly, vehicle formulation could include components that promote or reduce transdermal permeation, modulating efficacy and irritation of bioactives [99].
Site of application	Facial skin Eye contour Eyelid Body skin Hands Scalp Cuticle Legs	Safety	Although cosmeceuticals are intended to be applied to the skin, there are significant differences in the structure of the skin depending on the anatomical site. Therefore, the formulation of the vehicle must consider differences such as thickness, amount of lipids and water, hair density, and degree of sun exposure, among others.
In vitro permeation of cosmeceutical (from whole formulation)	Skin permeation rate Skin penetration depth	Efficacy Safety	In vitro release testing evaluates the ability of a formulation to deliver cosmeceuticals at the appropriate rate and depth at the site of application, contributing to building the safety–efficacy profile of the product [100].
Stability	At least 12 months shelf life at room temperature	Quality Efficacy	Stability involves the assessment of physical and chemical changes and proper microbiological preservation. The cosmetic product must maintain quality and functionality standards when stored under appropriate conditions.
pH	Near to skin pH 5.5	Efficacy Safety	The pH of the vehicle should be adjusted near to the skin physiological pH to avoid irritation; also, pH should be adjusted to assure chemical stability of the cosmeceutical and the physical performance of the vehicle during its shelf life.
Rheological/Textural profile	Viscosity Yield stress value G modulus value Distance of penetration value Force of penetration value Bioadhesion force value	Quality	Rheological and textural characteristics have an impact on cosmeceutical release from the nanocarrier and in skin retention of nanosystems [101]. In addition, rheological properties influence physical stability and sensorial profile.
Sensorial profile	Absorption Spreadability Pick-up Stickiness Brightness Oily	Quality	The sensory attributes of cosmetic products are largely decisive for the acceptance or rejection of the product by consumers.
Microbiological innocuity	Less than 100 CFU/g of aerobic mesophilic microorganism and absence of *Staphylococcus aureus*, *Pseudomonas aeruginosa*, *Candida albicans*, and *Escherichia coli*	Safety	Microbiological innocuity ensures that cosmetic products are safe for use and have been produced under good manufacturing practices.

**Table 3:** Examples of elements of QTPP that are related to the nanoparticles in which cosmeceuticals are included.

QTPP Element	Example of Target Parameter That Could Be Used	Characteristic Impacted	Considerations
Type of material	Inorganic, polymeric, lipid	Safety Efficacy	The excipients play an important role in the physicochemical characteristics of nanoparticles. The selection of the appropriate material is key for providing controlled release, bioadhesion, skin retention, compatibility bioactive-matrix, protection, biodegradability, etc.
Particle size	Hydrodynamic diameter	Quality Efficacy Safety	The particle size is one of the most relevant parameters to optimize, as it can affect nanoparticle skin penetration, adherence, clearance, and degradation [33]. For example, inorganic nanoparticles, at a size of 300–600 nm, were shown to penetrate and accumulate deeply into the hair follicles [102].
Size distribution	Polydispersity index	Quality	The PDI is an estimation of the uniformity of the nanoparticle size distribution; the lower the PDI, the greater the monodispersity. It could be considered an indicative parameter to control quality batch-by-batch during manufacturing.
Zeta potential	>30 mv	Quality	The zeta potential is a measure of the surface charge of nanoparticles, and this value defines their physical stability when they are suspended in an aqueous medium. It is considered that a value greater than 30 mV, whether positive or negative, is indicative of excellent stability, since there is sufficient electrostatic repulsion so that the particles do not aggregate [102]. On the other hand, the charge exhibited by the nanoparticles also participates in the interaction with the stratum corneum and in the retention of nanoformulations on the skin.
Entrapment or encapsulation efficiency	% *w*/*w*	Efficacy	The entrapment or encapsulation efficiency (EE%) in nanoparticle preparation is a measure of the total cosmeceutical added minus the free or the non-entrapped cosmeceutical over the total drug added [103]. The physicochemical characteristics of the cosmeceutical (Log P, ionization, charge, polarity, etc.) determine the %EE, in addition to factors inherent to the preparation method, such as solvent extraction method or pH conditions.
Loading capacity	Mass, % *w*/*w*	Efficacy	Loading capacity is defined as the exact amount of cosmeceutical that is included in the nanoparticles as dry mass. The value depends on the physicochemical properties and the structure of the carrier material [104].

**Table 4:** Phases of risk assessment in QbD.

Phase	Target	Tools
Risk identification	Identification of potential CPPs and CMAs	Ishikawa diagram (fishbone diagram), Preliminary Hazard Analysis
Risk analysis	Analysis of potential failure modes in a process, their causes, and effects	Failure Mode and Effects Analysis (FMEA), Fault Tree Analysis (FTA)
Risk evaluation	Provides a quantifiable evaluation of the occurrence, detectability, and severity of failure modes	Risk Matrix, Risk Ranking, Risk Priority Number

**Table 5:** Failure Mode and Effects Analysis (FMEA) of nanocosmeceutical products involving nanomaterials.

Failure Mode	Potential Causes	Potential Effects	Risk Mitigation
Excessive skin penetration and systemic absorption	Particles < 100 nm that can reach functional layers of the dermis	Skin irritation, toxicity, systemic effects	Specify the particle size range (e.g., 150–350 nm) and conduct a Dynamic Light Scattering (DLS) analysis. Perform an absorption analysis using Franz cells.
Instability or aggregation of nanoparticles in formulation	Lack of steric or electrostatic stabilization, pH shifts, inadequate storage conditions, mechanical stress during process, Ostwald ripening	Reduced efficacy, unpredictable release, aesthetic issues	Zeta potential and particle size analysis during stability test under stress conditions, during and after unit operations involved mechanical stress, excipient compatibility studies.
Loss of cosmeceutical load	Accelerated dissolution in vehicle, physical or chemical changes in bioactive, matrix nanoparticle degradation	Reduced efficacy	Accelerated stability testing, excipient compatibility studies.
Inconsistent manufacturing (batch variability)	Raw material variability, impurities	Variable efficacy, regulatory non-compliance	Quality assurance, process controls, implementation of PATs.
Occupational exposure	Formation of aerosols or dust	Production stoppages	Implement containment assessment by using local exhaust ventilation, wearing respirators, and conducting environmental monitoring.

**Table 6:** Potential set-up of DoEs applied to the design space step in nanocosmeceutical development.

Screening Designs	Optimization Design	Input Variables	Output Variables	Representation of Results	Validation of the Model
Two-level full factorial Fractionate factorial Plackett–Burman Simplex	Box–Behnken Central composite 3-level factorial Simplex centroid	Concentration of bioactive Agitation rate Time of agitation Membrane pore size % of components Polymer inherent viscosity Point of melt of lipid	Particle size PDI Zeta potential Load capacity Bioactive content Penetration depth Skin irritation Viscosity pH Yield strength Stability (kinetic constant)	Pareto chart Main effects graph Surface graph Contours graph Ternary diagram Desirability plot	ANOVA analysis Multiple regression modeling Residual plot Levene’s test Normal plot Shapiro–Wilk’s test Kruskal–Wallis test

## Data Availability

No new data were created or analyzed in this study. Data sharing is not applicable to this article.

## Abbreviations

The following abbreviations are used in this manuscript:

ADME	Absorption, Distribution, Metabolism, and Excretion
AgNP	Silver Nanoparticles
AuNP	Gold Nanoparticles
CMAs	Critical Material Attributes
CFU	Colony Forming Unit
CQAs	Critical Quality Attributes
CpK	Capability Index
CPPs	Critical Process Parameters
DoE	Design of Experiments
FDA	Food and Drug Administration
ICH	International Council for Harmonisation
LNPs	Lipid Nanoparticles
NC	Nanocarrier
NEGs	Nanoemulsion Gels
NLCs	Nanostructured Lipid Carriers
OMC	Octyl Methoxycinnamate
PAT	Process Analytical Technology
PNP	Polymeric Nanoparticles
QbD	Quality by Design
QTPP	Quality Target Product Profile
SC	Stratum Corneum
SLNs	Solid Lipid Nanoparticles
USFDA	United States Food and Drug Administration
